# Efficacy and safety of thrombopoietin receptor agonists in solid tumors with chemotherapy-induced thrombocytopenia: a meta-analysis

**DOI:** 10.1186/s40360-023-00707-5

**Published:** 2023-12-01

**Authors:** Wen Chen, Yubingxue Liu, Luchun Li, Xianghua Zeng

**Affiliations:** 1 Department of Medical Oncology, Chongqing University Cancer Hospital, Chongqing, 400030 China; 2 Department of Health Examination and Oncology Screening Center, Chongqing University Cancer Hospital, Chongqing, 400030 China

**Keywords:** Solid tumors, Thrombopoietin receptor agonists, Chemotherapy-induced thrombocytopenia, Meta-analysis, Randomized controlled trials

## Abstract

**Objective:**

To evaluate the efficacy and safety of thrombopoietin receptor agonists (TPO-RAs) in solid tumors with chemotherapy-induced thrombocytopenia (CIT).

**Methods:**

We conducted a comprehensive search of PubMed, FMRS, Cochrane Library, Web of Science, EMBASE, and ClinicalTrials.gov for randomized controlled trials (RCTs) reporting the efficacy and safety of TPO-RAs in solid tumors with CIT. The search was limited to articles published before April 30, 2022. Primary outcomes included chemotherapy dose reduction or delays, platelet transfusion, the incidence of grade 3 or 4 thrombocytopenia, and bleeding events. Secondary outcomes encompassed the incidence of platelet count > 400 × 10^9^/L, adverse events (AEs), serious AEs, thrombosis, and mortality.

**Results:**

Our analysis encompassed six studies: five rigorous RCTs and one unique study comparing romiplostim to an observation group, involving a total of 489 patients. For primary outcomes, TPO-RAs significantly reduced the incidence of grade 3 or 4 thrombocytopenia (RR = 0.69, 95% CI: 0.52–0.91). After applying the Bonferroni correction for multiple comparisons, the significance of the reduction in grade 3 or 4 thrombocytopenia incidence persisted (*P* = 0.008). TPO-RAs showed no significant impact on chemotherapy dose reduction or delays (RR = 0.81, 95% CI: 0.65–1.01), platelet transfusion (RR = 1.04, 95% CI: 0.48–2.27), or bleeding events (RR = 0.50, 95% CI: 0.23–1.10). In terms of safety, there were no significant difference in the incidence of any AEs (RR = 0.98, 95% CI:0.92–1.04), serious AEs (RR = 0.79, 95% CI:0.45–1.40), thrombotic events (RR = 1.20, 95% CI:0.51–2.84) and mortality (RR = 1.15, 95% CI:0.55–2.41).

**Conclusions:**

This meta-analysis suggests that TPO-RAs are generally well-tolerated. However, their efficacy in solid tumors with CIT appears limited, as they only demonstrate a reduction in the incidence of grade 3 or 4 thrombocytopenia.

**Supplementary Information:**

The online version contains supplementary material available at 10.1186/s40360-023-00707-5.

## Introduction

Chemotherapy-induced thrombocytopenia (CIT) is a common complication of cancer treatment. It leads to chemotherapy delays, dose reductions, and treatment discontinuation, negatively impacting treatment outcomes and increasing the risk of bleeding for patients [1]. Currently, there is no agent approved by the US Food and Drug Administration (FDA) for the treatment of CIT. The only standard approach to managing CIT is platelet transfusion [2]. However, platelet transfusion provides only temporary improvement, and it is impossible to sustain over extended periods. Therefore, chemotherapy dose reductions and treatment delays are usually inevitable, which may decrease relative dose intensity and reduce the efficacy of chemotherapy [3].

The thrombopoietin receptor agonists (TPO-RAs) are a class of platelet growth factors, including eltrombopag, avatrombopag, romiplostim and lusutrombopag. TPO-RAs bind to the thrombopoietin receptor. This binding causes a conformational change in the thrombopoietin receptor, activates the JAK2/STAT5 pathway, and increases megakaryocyte progenitor proliferation and platelet production. Currently, TPO-RAs have been FDA-approved for immune thrombocytopenia in cases of insufficient response to pretreatment, periprocedural thrombocytopenia in patients with chronic liver disease, aplastic anemia, and thrombocytopenia associated with antiviral treatment of hepatitis C [4, 5].

TPO-RAs represent a new potential therapy for the CIT. In previous phase 1 and 2 clinical trials, it was shown that compared with placebo, eltrombopag increased platelet count during chemotherapy in solid tumors [6, 7]. In a phase 2 clinical trial of romiplostim in the treatment of solid tumors with CIT showed that during the initial randomized phase, 14 of 15 romiplostim-treated patients (93%) experienced a restoration of their platelet count within 3 weeks, compared with only one of eight control patients (12.5%). Because of the promising results observed in the romiplostim arm, the study was converted to a single-arm trail, and 44 out of 52 patients (85%) who achieved platelet correction with romiplostim resumed chemotherapy with weekly romiplostim, thus demonstrating the effectiveness of romiplostim in the treatment of CIT [8]. However, in a phase 3 clinical trial of avatrombopag in the treatment of solid tumors with CIT, there was no significant improvement in the proportion of patients meeting the composite primary endpoint (i.e., the proportion of responders who did not require platelet transfusion, chemotherapy dose reduction or chemotherapy delays) between the experimental group and the placebo group [9].

Given that TPO-RAs may be an important treatment for CIT in solid tumors, this study aims to conduct a meta-analysis of the published data to evaluate the efficacy and safety of TPO-RAs in solid tumors with CIT.

## Materials and methods

### Protocol and registration

This systematic review was conducted in accordance with the PRISMA Extension Statement for Reporting of Systematic Reviews Incorporating Network Meta-analyses of Health Care Interventions [10]. It was registered under the registration numbers CRD42023461834 on the PROSPERO website.

### Search strategy

PubMed, FMRS, Cochrane Library, Web of Science, EMBASE and ClinialTrials.gov were systematically searched to identify potentially eligible studies. The search was limited to articles published before April 30, 2022, and to English-language publications. The search terms and MeSH(Medical Subject Headings) primarily included “thrombopoietin receptor agonists”, “Chemotherapy”, “thrombocytopenia” and “clinical trial”. Details of the study selection process are provided in supplementary materials.

### Study selection

Two reviewers (CW and LYBX) independently screened the titles and abstracts of all studies for eligibility, and the records that seemed likely to meet the inclusion criteria were retrieved in full text. The following types of studies were excluded: reviews and systematic reviews, non-human studies, case reports, observational research, cohort studies, retrospective analysis, pharmacokinetics and articles unrelated to the topic of this study. Differences of opinion between reviewers were resolved through discussion or by a third party.

### Inclusion and exclusion criteria

Inclusion criteria: (1) Research design: randomized controlled trials; (2) Patients: solid tumors patients with CIT older than 18 years old; (3) Interventions: Eltrombopag or Romiplostim or Avatrombopag compared with placebo or blank; (4) Outcome indicators: incidence of chemotherapy dose reduction or delays, bleeding events, platelet transfusion, incidence of grade 3 or 4 thrombocytopenia, incidence of platelet count > 400 × 10^9^/L, adverse events(AEs), serious AEs, embolism events and deaths. Exclusion criteria: (1) Conference abstracts; (2) Information on the trial was missing or incomplete.

### Outcome measure

The primary outcomes were chemotherapy dose reduction or delays, platelet transfusion, the incidence of grade 3 or 4 thrombocytopenia and bleeding events. To control the potential risk of false positives with multiple comparisons, we performed Bonferroni correction. Secondary outcomes included the incidence of platelet count > 400 × 10^9^/L, AEs, serious AEs, thrombosis and mortality.

### Date extraction

Two authors (CW and LYBX) extracted the data independently to complete the extraction table, disagreements between authors were resolved by discussion or decided by the third party. The date included in the extraction table were as follows: (1) first author’s name, publication time, regions and registration number of trails, randomization, total number of participants; (2) age and gender of the patients; (3) intervention characteristics (type, dose, and duration); (4) outcome indicators: bleeding events, platelet transfusion, chemotherapy dose reduction or delays, incidence of grade 3 or 4 thrombocytopenia, incidence of platelet count > 400 × 10^9^/L; (5) safety data: AEs, serious AEs, thrombosis and mortality.

### Evidence quality assessment

The risk of bias was assessed using Cochrane’s risk of bias tool for RCTs (RoB v2.0) [11]. The assessment of risk of bias was conducted independently by two reviewers (CW and LYBX) across five domains (randomization process, deviations from intended interventions, missing outcome data, measurement of the outcome and selection of the reported result). Ratings for bias were categorized as “low risk,“ “some concerns,“ or “high risk.“ Disagreements were resolved through consultation with a third evaluator (LLC).

### Statistical analyses

The outcome data in this study consisted of dichotomous variables. We pooled trials using meta-analysis with RevMan5.3, applying a random-effects model to assess the overall estimated effects. Risk ratios (RR) with 95% confidence intervals (CI) were employed for evaluating dichotomous variables. We tested Heterogeneity using the I^2^ statistic and the Cochran Q-test. A significance level of I^2^ ≥ 50% and *P* < 0.10 was considered indicative of significant heterogeneity. When significant heterogeneity was observed, we conducted sensitivity and subgroup analyses to provide possible explanations. To account for multiple testing in the meta-analysis, we applied Bonferroni adjustment, resulting in a rejection P-value of 0.05 divided by the total number of outcomes. In this meta-analysis, there were four primary outcomes; thus, the rejection P-value was calculated as 0.05/4 = 0.0125.

## Results

### Study selection and characteristics

**Fig. 1:**
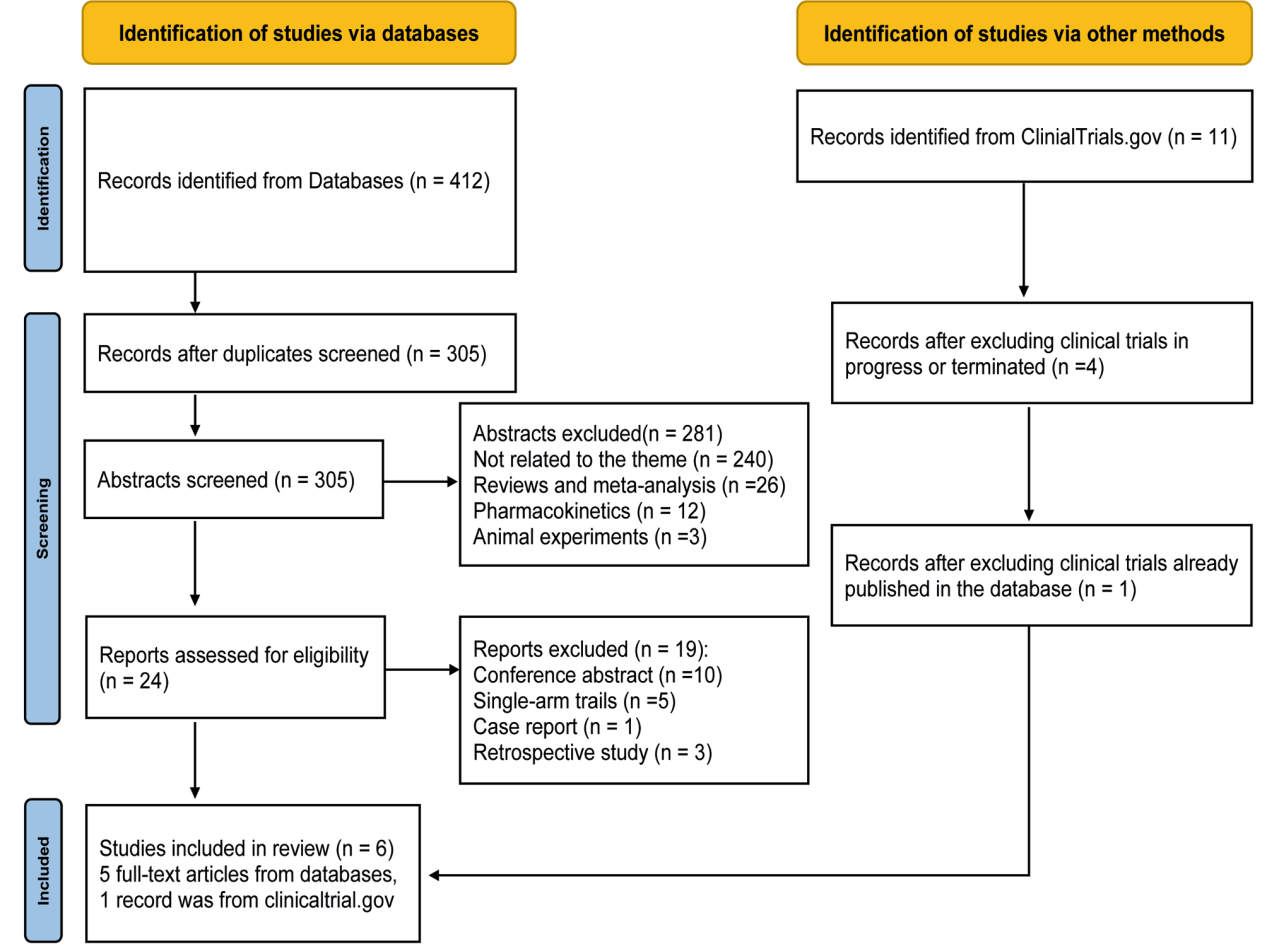
Study flow diagram

A total of 423 potential records were retrieved. Among these, 39 came from PubMed, 124 from the Cochrane library, 79 from EMBASE, 45 from FMRS, 125 from Web of Science, and 11 from ClinialTrials.gov. After excluding 107 duplicates, 240 irrelevant articles, 26 reviews and systematic reviews, 12 pharmacokinetics, 3 animal experiments, we identified 24 records. Subsequently, we carefully screened these 24 articles. We excluded 5 articles because they were single-arm trails, 3 articles because they were retrospective studies, and 10 conference abstracts and 1 case report. Our search on ClinicalTrials.gov yielded 11 studies, of which 7 were clinical trials in progress or terminated, and 4 had already been published in the database, duplicating the literature retrieved. Therefore, we excluded these 4 trials. In the end, our study included 5 articles and 1 clinical trial, encompassing 489 participants. The flow diagram of the literature search is presented in Fig. 1.

Six trials were included [6–9, 12, 13], of which 3 compared eltrombopag to placebo [6, 7, 13], one compared avatrombopag to placebo [9], one compared romiplostim to placebo [12] and one compared romiplostim to the observation group [8]. 5 studies were randomized double-blind controlled studies [6, 7, 9, 12, 13], and one study was a randomized open controlled study [8]. The study included a total of 489 patients, with 353 in the experimental group and 136 in the control group. One trial was a phase 1 clinical trial [6], four were phase 2 clinical trials [7, 8, 12, 13], and one was a phase 3 clinical trial [9].The characteristics of the clinical trials in this meta-analysis are presented in Table 1.

**Table 1:** Characteristics of the included studies

Author and Year, Tumor type, Registration Number, Stage	Regions	Age(years)	Gender (male,%)	Interventions	Participants	Random and blind method	outcomes	Period of study
Natale R (2009), Non-Small Cell Lung Cancer, NCT00413283, Phase 2	USA, Europe, Canada, Austria	63.8 ± 10.8	75%	Romiplostim 250 µg	N = 16	1:1:1:1Random, double-blind	Chemotherapy dose reduction or delays, grade 3 or 4 thrombocytopenia, platelet transfusion, AEs, serious AEs	4 months
		62.5 ± 7.7	66.7%	Romiplostim 500 µg	N = 18			
		65.4 ± 8.2	88.2%	Romiplostim 750 µg	N = 17			
		59.8 ± 6.6	50%	Placebo	N = 12			
Winer(2015), Solid tumors, NCT01147809, Phase 1	USA, Europe, India	55.0(34.0–74.0)	47%	Eltrombopag 100 mg	N = 19	3:1Random, double-blind	Chemotherapy dose reduction or delays, grade 3 or 4 thrombocytopenia, platelet count > 400 × 10^9^/L, AEs, serious AEs, thrombosis, mortality	6 cycles of chemotherapy
		61.0(31.0–81.0)	43%	Placebo	N = 7			
Winer(2017), Solid tumors, Not given, Phase 2	USA, Europe	67.0(36.0–82.0)	55.8%	Eltrombopag 100 mg	N = 52	2:1Random, double-blind	Bleeding events, platelet transfusion, chemotherapy dose reduction or delays, grade 3 or 4 thrombocytopenia, AEs, serious AEs, thrombosis	6 cycles of chemotherapy
		66.0(44.0–83.0)	43.5%	Placebo	N = 23			
Al-Samkari(2022), Solid tumors, NCT03471078, Phase 3	USA, Europe、China	62.0(52.0–69.0)	48%	Avatrombopag 60 mg	N = 82	2:1Random, double-blind	Bleeding events, platelet transfusion, chemotherapy dose reduction or delays, grade 3 or 4 thrombocytopenia, platelet count > 400 × 10^9^/L; AEs, serious AEs, thrombosis, mortality.	Not given
		63.5(54.0–67.0)	45%	Placebo	N = 40			
Mantha(2019), Solid tumors, NCT02052882, Phase 2	USA	50.0(30.0–76.0)	30%	Romiplostim	N = 15	2:1Random, open	Platelet transfusion, AEs, serious AEs	3 weeks after enrollment
		67.0(46.0–77.0)	75%	Observation group	N = 8			
Kellum(2010), Solid tumors, NCT00102726, Phase 2	USA, European Union, Asia, South America	58.5(35.0–75.0)	52%	Eltrombopag 50 mg	N = 44	1:1:1:1Random, double-blind	Bleeding events, platelet count > 400 × 10^9^/L; AEs, thrombosis, mortality	Not given
		59.0 (33.0–75.0)	36%	Eltrombopag 75 mg	N = 44			
		58.0 (34.0–81.0)	52%	Eltrombopag 100 mg	N = 46			
		58.0(23.0–73.0)	35%	Placebo	N = 46			

### Risk of bias

**Fig. 2:**
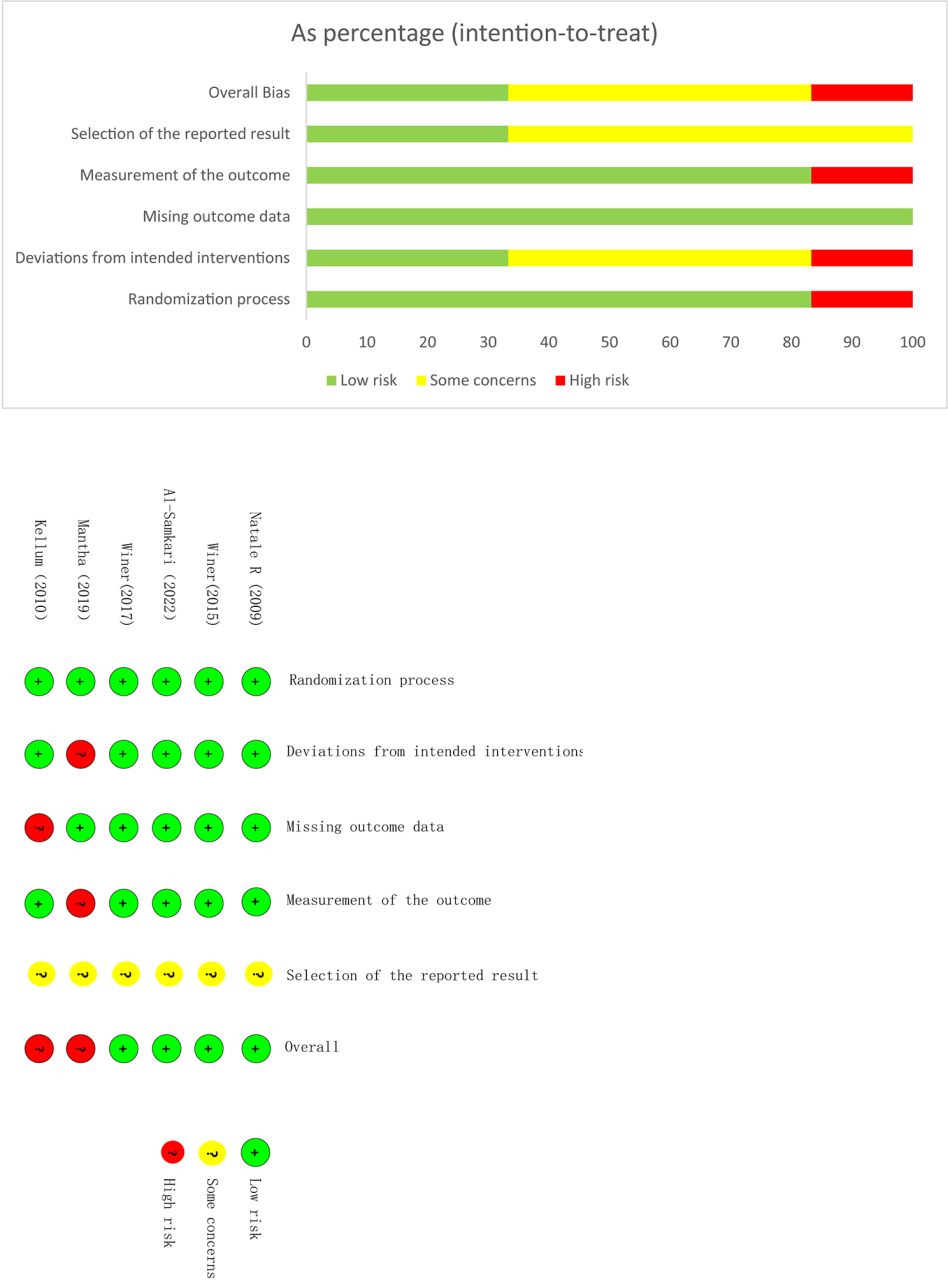
Risk of bias graph and Risk of bias summary

We utilized the Cochrane Collaboration’s tool to assess the quality of the included RCTs. Despite their small size, these trails were deemed to be of high quality (see Fig. 2). Among the five randomized double-blind controlled studies, detailed information about the generation of random sequences and allocation concealment methods was provided. However, the single randomized open study may carry a risk of selective reporting (see Fig. 2).

### Efficacy outcomes

#### Incidence of chemotherapy dose reduction or delays, platelet transfusion, bleeding events

**Fig. 3:**
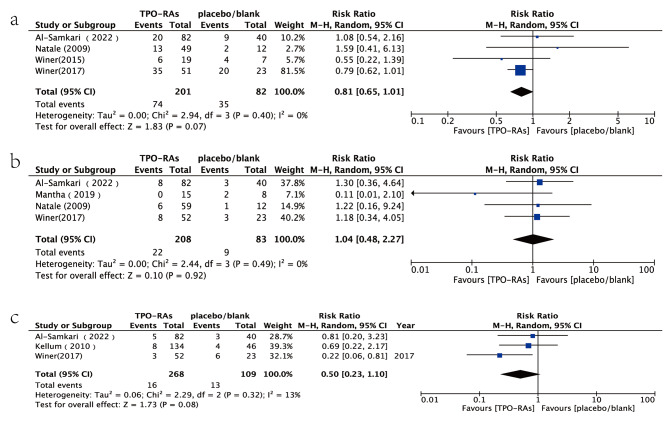
Forest plot and meta-analysis of visual clarity. (**a**) Incidence of chemotherapy dose reduction or delays. (**b**) Requirement of platelet transfusion. (**c**) Incidence of hemorrhagic events

Four studies assessed chemotherapy dose reduction or delays as outcome indicator [6, 7, 9, 12]. In both chemotherapy groups, there were fewer instances of chemotherapy dose reduction or delays in patients receiving TPO-RAs (36.81%) compared to those receiving a placebo (42.68%). However, this difference was not statistically significant (RR = 0.81,95% CI:0.65–1.01, *P* > 0.05). Additionally, four studies used platelet transfusion as an outcome indicator [7–9, 12]. There was no significant difference in the proportion of platelet transfusion between the experimental and control group (RR = 1.04, 95% CI:0.48–2.27, *P* > 0.05). Furthermore, three studies used bleeding events as outcome indicator [7, 9, 13]. The incidence of bleeding events in experimental group (5.97%) was lower than that in the control group (11.93%), but this difference was not statistically significant (RR = 0.50; 95% CI:0.23–1.10, *P* > 0.05) (Fig. 3).

#### Incidence of grade 3 or 4 thrombocytopenia and platelet count > 400 × 10^9^/L

**Fig. 4:**
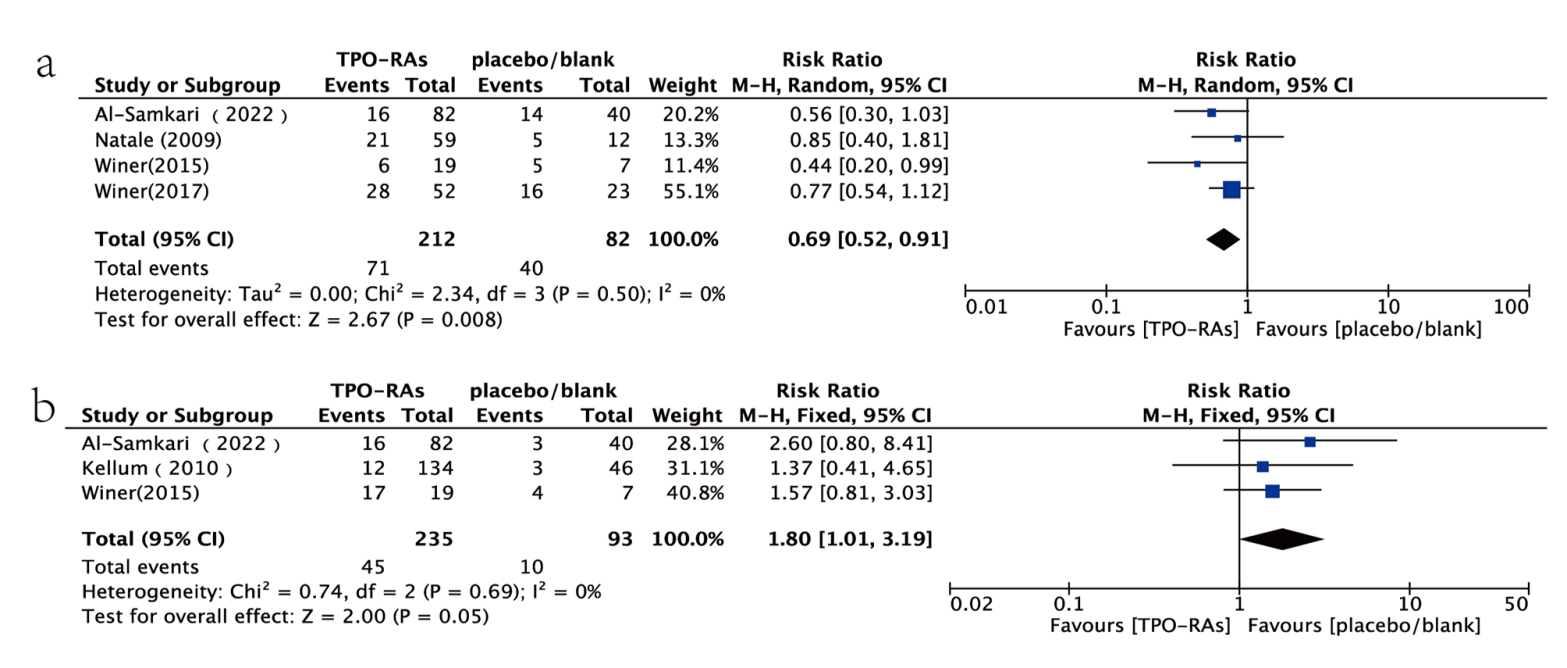
Forest plot and meta-analysis of visual clarity. (**a**) Incidence of grade 3 or 4 thrombocytopenia. (**b**) Occurrences of platelet count > 400 × 10^9^/L

Four studies compared the incidence of grade 3 or 4 thrombocytopenia [6, 7, 9, 12]. There was a lower incidence of grade 3 or 4 thrombocytopenia in patients receiving TPO-RAs (33.49%) compared to those receiving a placebo (48.78%) in both chemotherapy groups (RR = 0.69, 95% CI:0.52–0.91, *P* < 0.05). Additionally, three studies used the incidence of platelet count > 400 × 10^9^/L as an outcome indicator [6, 9, 13]. The incidence of platelet count > 400 × 10^9^/L in the experimental group (19.14%) was higher than that in the control group (10.75%), and this difference was statistically significant (RR = 1.80, 95% CI:1.01–3.19, *P* < 0.05) (Fig. 4). After applying Bonferroni correction, with an adjusted significance level of 0.0125 (0.05 divided by the 4 primary endpoints), only the results related to the incidence of grade 3 or 4 thrombocytopenia remained statistically significant.

### Safety outcomes

#### Incidence of any AEs, serious AEs, thrombosis and mortality

**Fig. 5:**
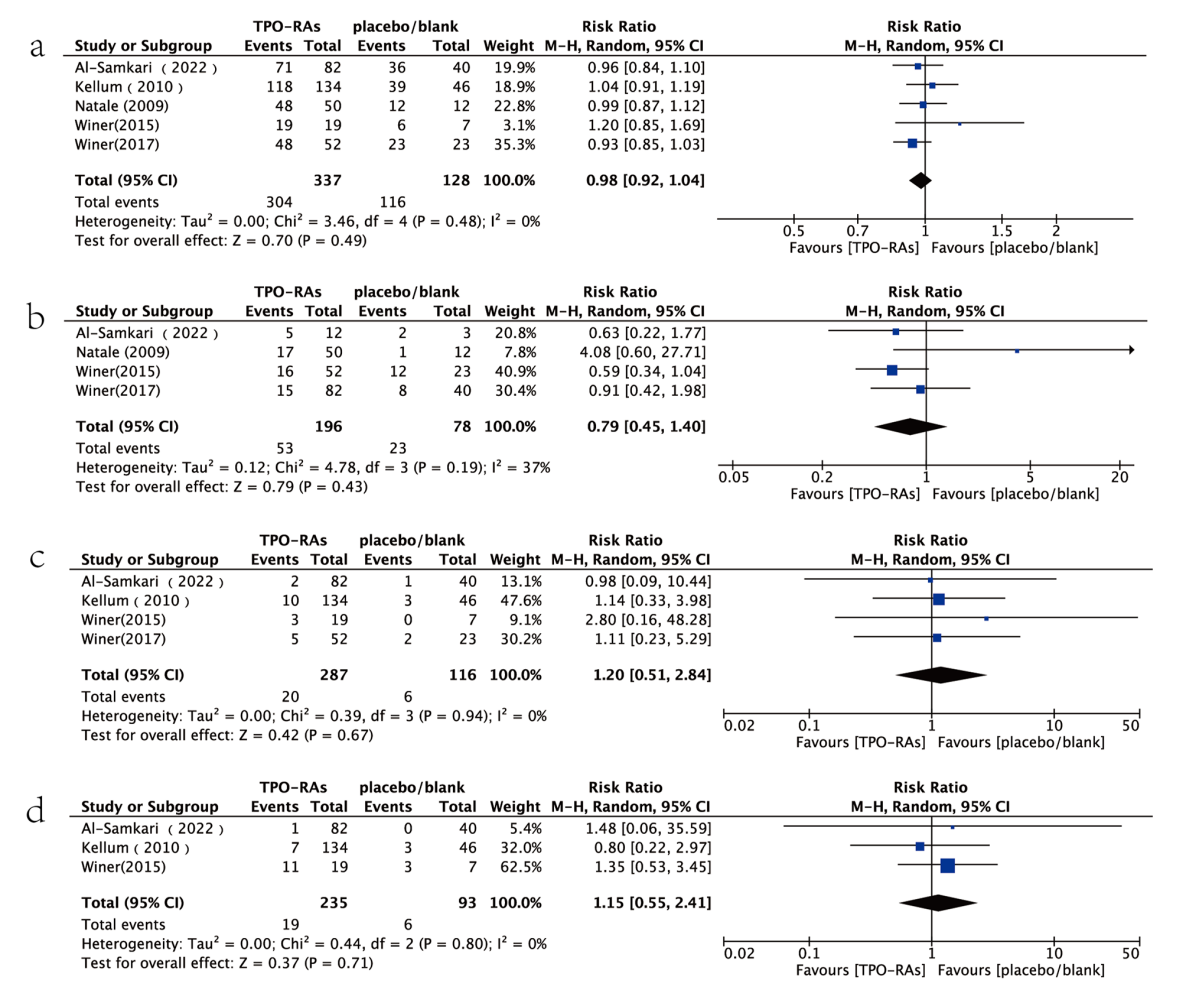
Forest plot and meta-analysis of visual clarity. (**a**) Incidence of any AEs. (**b**) Incidence of serious AEs. (**c**) Incidence of thrombosis. (**d**) Incidence of mortality

Five studies compared the incidence of any AEs [6, 7, 9, 12, 13]. The incidence of any AEs in the experimental group was similar to that in the control group (RR = 0.98,95% CI:0.92–1.04, *P* > 0.05). Furthermore, four studies evaluated the incidence of serious AEs [6, 7, 9, 12]. There was no significant difference in the proportion of serious AEs between the experimental and control group (RR = 0.79, 95% CI: 0.45–1.40, *P* > 0.05). Additionally, four studies compared the incidence of thrombosis [6, 7, 9, 13]. The incidence of thrombosis in the experimental group (6.97%) was similar to that in the control group (5.17%) (RR = 1.20, 95% CI:0.51–2.84, *P* > 0.05). Finally, three studies evaluated the incidence of mortality [6, 9, 13]. The incidence of mortality in the experimental group (8.09%) was higher than that in the control group (6.45%), but the difference was not statistically significant (RR = 1.15, 95% CI:0.55–2.41, *P* > 0.05) (Fig. 5).

## Discussion

Approximately 10–38% of patients with solid tumors who undergo chemotherapy develop CIT [14]. The incidence and prevalence of CIT vary significantly depending on the type of chemotherapy regimens. For example, gemcitabine-based and platinum‐based regimens consistently carry the highest risk of thrombocytopenia [3]. Currently, there are no standardized guidelines for the prevention or treatment of CIT. When the platelet count drops too low, guidelines recommend prophylactic platelet transfusion to prevent and treat bleeding. However, platelet transfusion has several disadvantages, including high cost, a short effective time, and the risk of allergy. In terms of drugs, first-generation recombinant thrombopoietin initially showed promise in reducing chemotherapy-related thrombocytopenia in early clinical trials. However, their further development was halted due to the development of antibody against endogenous thrombopoietin [3]. Another approach involves using recombinant human interleukin-11 (rhIL-11), which has received FDA approval for the treatment of CIT in non-myeloid tumors. Nevertheless, pharmacoeconomic disadvantages and adverse effects such as cardiotoxicity and edema have limited its use [15].

TPO-RAs are second-generation thrombopoietin receptor agonists that mimic the function of endogenous thrombopoietin without inducing cross-reactive antibodies. From preclinical studies [16] to clinical trials, TPO-RAs have demonstrated efficacy and safety in the treatment of CIT. To date, five published clinical trials have reported the use of TPO-RAs in CIT [6–9, 13]. In all of these studies, TPO-RAs were found to be well-tolerated. However, it is not possible to directly compare the efficacy of various TPO-RAs in the treatment of CIT due to differences in the design of clinical trials.

A change in platelet count serves as a direct indicator to assess the efficacy of TPO-RAs. Unfortunately, our study was not originally designed to measure this indicator as the required data could not be extracted. Only one study reported platelet response [8]. Another study reported the nadir platelet count during chemotherapy [6]. Additionally, one study reported the mean increase in platelet count from the nadir [9]. Furthermore, three studies reported the mean platelet count on day 1 before chemotherapy, with one study reporting the day 1 prechemotherapy count across all cycles [7], and two studies reporting the day 1 prechemotherapy for the second cycle [9, 13]. Therefore, the available data were not suitable for direct comparison. It is worth noting that in three studies, the experimental group exhibited a significantly higher platelet elevation compared to the control group [6, 8, 9].

In our study, the main outcomes used to assess efficacy included the incidence of chemotherapy dose reduction or delays, platelet transfusion, and bleeding events. We found that the incidence of chemotherapy dose reduction or delays in the experimental group was similar to that in the control group, although we observed a trend of lower chemotherapy dose reduction or delays in the experimental group. Similarly, bleeding events and the need for platelet transfusion in the experimental group were comparable to those in the control group. These results suggest that TPO-RAs did not demonstrate a clear advantages over the control group in terms of the main outcome indicators of efficacy. Notably, achieving a platelet count greater than 400 × 10^9^/L may be considered one of the manifestations of drug’s effect. In this regard, three studies counted the number of cases with a platelet count exceeding 400 × 10^9^/L, and the proportion in the experimental group was significantly higher than that in the control group [6, 9, 13]. In addition, grade 3 or 4 thrombocytopenia served as an indicator of treatment efficacy. In comparison with the control group, the incidence of grade 3 or 4 thrombocytopenia in the experimental group was lower, suggesting that TPO-RAs helped prevent the occurrence of severe thrombocytopenia.

Bonferroni correction was applied to account for multiple comparisons in the analysis of our primary endpoints. This correction method is widely used in research to control the potential risk of false positives when multiple comparisons are made simultaneously. The application of Bonferroni correction is a conservative approach that helps mitigate the inflation of Type I error rates. After applying this correction in our analysis, we found that only the results related to the incidence of grade 3 or 4 thrombocytopenia remained statistically significant. This outcome underscores the robustness of our finding.

For safety, no statistical differences were found between the experimental and control groups in any AEs or serious AEs. There was also no significant difference in all-cause mortality between the two groups. These results indicate that TPO-RAs in CIT were safe and tolerable.

Thrombosis was considered as a separate safety event for follow reasons: first, cancer patients undergoing chemotherapy are at a high risk of thrombosis [17]; second, the proportion of platelets > 400 × 10^9^/L in the experimental group is higher, and an elevated platelet count is also a risk factor for thrombosis [18]; third, TPO-RAs have been reported to increase the risk of thrombosis, but the mechanism remains unclear [19]. According to our analysis, there was no significant difference in the incidence of thrombosis between the experimental and control group, indicating that TPO-RAs did not increase the risk of thrombosis in CIT.

As far as we know, the present meta-analysis has included the largest number of studies and performed the most comprehensive analysis. Previous researchers have done similar analysis, but those studies did not yield referenceable conclusions. Nonetheless, our research has several limitations. First, the conclusions were based on a limited number of studies, with a relatively small number of events. Second, experimental designs were different among studies, and some required data could not be extracted. Third, one study was random open-label, with the risk of selective reporting. Fourth, we did not perform subgroup analysis according to different types and doses of drugs for limited studies. Therefore, large-scale, rigorously designed, multi-center randomized clinical trials are needed to expand the data.

## Conclusion

In summary, this meta-analysis demonstrates that TPO-RAs are tolerable and can reduce grade 3 or 4 thrombocytopenia in solid tumors with CIT. However, they do not confer advantages in terms of the main outcomes used to assess efficacy, including chemotherapy dose reduction or delays, platelet transfusion, and bleeding events. While TPO-RAs show promise as a potential therapeutic option for solid tumors with CIT, current research results indicate limited efficacy.

### Electronic supplementary material

Below is the link to the electronic supplementary material.

Supplementary Material 1

Supplementary Material 2

Supplementary Material 3

## Data Availability

The datasets used and analyzed during the current study are available from the corresponding author on reasonable request.
