# Supplementary material for: Efficacy and safety of thrombopoietin receptor agonists in solid tumors with chemotherapy-induced thrombocytopenia: a meta-analysis
Source: BMC Pharmacol Toxicol. 2023 Dec 1;24:71. doi: 10.1186/s40360-023-00707-5 (PMC10693054; doi:10.1186/s40360-023-00707-5)
Supplement: Supplementary file 2 — Supplementary Material 2 [file 40360_2023_707_MOESM2_ESM.pdf]

This PDF document provides information on our Prospero registration.

Systematic review

A list of fields that can be edited in an update can be found here

1. * Review title.

Give the title of the review in English

Efficacy and Safety of Thrombopoietin Receptor Agonists in Solid Tumors with Chemotherapy-induced Thrombocytopenia

2. Original language title.

For reviews in languages other than English, give the title in the original language. This will be displayed with the English language title.

3. * Anticipated or actual start date.

Give the date the systematic review started or is expected to start.

08/09/2023

4. * Anticipated completion date.

Give the date by which the review is expected to be completed.

08/10/2023

5. * Stage of review at time of this submission.

This field uses answers to initial screening questions. It cannot be edited until after registration.

Tick the boxes to show which review tasks have been started and which have been completed.

Update this field each time any amendments are made to a published record.

The review has not yet started: No

Review stage	Started	Completed
Preliminary searches	Yes	No
Piloting of the study selection process	Yes	No
Formal screening of search results against eligibility criteria	Yes	No
Data extraction	No	No
Risk of bias (quality) assessment	No	No
Data analysis	No	No

Provide any other relevant information about the stage of the review here.

6. * Named contact.

The named contact is the guarantor for the accuracy of the information in the register record. This may be any member of the review team.

Wen Chen

Email salutation (e.g. "Dr Smith" or "Joanne") for correspondence:

Mr Chen

7. * Named contact email.

Give the electronic email address of the named contact.

836049985@qq.com

8. Named contact address

Give the full institutional/organisational postal address for the named contact.

181 Han-Yu Road, Shapingba District, Chongqing City, China

9. Named contact phone number.

Give the telephone number for the named contact, including international dialling code.

17830080060

10. * Organisational affiliation of the review.

Full title of the organisational affiliations for this review and website address if available. This field may be

completed as 'None' if the review is not affiliated to any organisation.

Chongqing University Cancer Hospital

Organisation web address:

11. * Review team members and their organisational affiliations.

Give the personal details and the organisational affiliations of each member of the review team. Affiliation refers to groups or organisations to which review team members belong. **NOTE: email and country now MUST be entered for each person, unless you are amending a published record.**

Mr wen Chen. Chongqing University Cancer Hospital
Mr Xianghua Zeng. Chongqing University Cancer Hospital
Mrs Yubingxue Liu. Chongqing University Cancer Hospital
Mrs Luchun Li. Chongqing University Cancer Hospital

12. * Funding sources/sponsors.

Details of the individuals, organizations, groups, companies or other legal entities who have funded or sponsored the review.

This study was supported by the Integrated innovation and application of key technologies for precise prevention and treatment of primary lung cancer (No.2019ZX002) and Natural Science Foundation of Chongqing, China (No.cstc2018jcyjAX0814).

Grant number(s)

State the funder, grant or award number and the date of award

13. * Conflicts of interest.

List actual or perceived conflicts of interest (financial or academic).

None

14. Collaborators.

Give the name and affiliation of any individuals or organisations who are working on the review but who are not listed as review team members. **NOTE: email and country must be completed for each person, unless you are amending a published record.**

15. * Review question.

State the review question(s) clearly and precisely. It may be appropriate to break very broad questions down into a series of related more specific questions. Questions may be framed or refined using PI(E)COS or similar where relevant.

Are thrombopoietin teceptor agonists more effective than placebo in elevating platelets in a population of patients with chemotherapy-induced thrombocytopenia?

16. * Searches.

State the sources that will be searched (e.g. Medline). Give the search dates, and any restrictions (e.g. language or publication date). Do NOT enter the full search strategy (it may be provided as a link or attachment below.)

PubMed, FMRS, Cochrane Library, Web of Science, EMBASE and ClinialTrials.gov were systematically searched to identify potentially eligible studies. The search was restricted to articles published before April 30, 2022. The search results were restricted to English publications. Search terms and MeSH mainly were "thrombopoietin receptor agonists", "Chemotherapy", "thrombocytopenia" and "clinical trial".

17. URL to search strategy.

Upload a file with your search strategy, or an example of a search strategy for a specific database, (including the keywords) in pdf or word format. In doing so you are consenting to the file being made publicly accessible. Or provide a URL or link to the strategy. Do NOT provide links to your search **results**.

Alternatively, upload your search strategy to CRD in pdf format. Please note that by doing so you are consenting to the file being made publicly accessible.

Yes I give permission for this file to be made publicly available

18. * Condition or domain being studied.

Give a short description of the disease, condition or healthcare domain being studied in your systematic review.

The disease studied in this systematic evaluation is chemotherapy-induced thrombocytopenia, a common post-chemotherapy side effect. Chemotherapy-induced thrombocytopenia may lead to increased risk of bleeding, delayed chemotherapy and increased healthcare burden. Our study is concerned with the ability of thrombopoietin receptor agonists to increase platelet counts in patients during treatment, thereby attenuating or preventing the adverse effects caused by thrombocytopenia.

19. * Participants/population.

Specify the participants or populations being studied in the review. The preferred format includes details of both inclusion and exclusion criteria.

Inclusion Criteria:

Our systematic review study will include the following types of participants or populations:

1. Patients with solid tumors: patients diagnosed with solid tumors, not limited to specific types of solid tumors.

2. Treated with chemotherapy: included patients must be treated with chemotherapy.

3. Intervention: use of at least one of eltrombopag, avatrombopag, romiplostim compared to placebo.

4. Age: we will include patients older than 18 years of age.

5. Type of literature: we will include randomized controlled trials (RCTs).

Exclusion criteria:

1. Patients not treated with chemotherapy.

2.Type of literature: We will exclude retrospective studies, case reports, expert opinion and other non-randomized controlled studies.

Our inclusion criteria are designed to ensure that the study population is relevant to our research question and can provide useful information about the efficacy and safety of thrombopoietin receptor agonists in chemotherapy-induced thrombocytopenia. We will screen the literature based on these criteria to ensure that the most relevant studies are included.

20. * Intervention(s), exposure(s).

Give full and clear descriptions or definitions of the interventions or the exposures to be reviewed. The preferred format includes details of both inclusion and exclusion criteria.

We will review pharmacotherapeutic interventions for chemotherapy-induced thrombocytopenia. These treatments include the following drugs: eltrombopag, avatrombopag, romiplostim. To be eligible for inclusion, studies had to include patients with chemotherapy-induced thrombocytopenia, treated with at least one of the above-mentioned drugs.

21. * Comparator(s)/control.

Where relevant, give details of the alternatives against which the intervention/exposure will be compared (e.g. another intervention or a non-exposed control group). The preferred format includes details of both inclusion and exclusion criteria.

We will compare the thrombopoietin receptor agonists therapeutic intervention with the following alternatives or Control groups:

1. Control group: patients receiving placebo treatment, that is, the non-intervention group.

2. Alternative control group: patients who did not receive any treatment, that is, patients who were not exposed to thrombopoietin receptor agonists or placebo treatment.

To be eligible for inclusion, studies had to clearly describe the intervention or control group patients received and meet specified criteria. We will exclude studies that do not meet these criteria to ensure consistency and comparability of the review.

22. * Types of study to be included.

Give details of the study designs (e.g. RCT) that are eligible for inclusion in the review. The preferred format includes both inclusion and exclusion criteria. If there are no restrictions on the types of study, this should be stated.

We only planned to include randomized controlled trials (RCTs). Other types of research designs will be excluded.

To be eligible for inclusion, studies must meet the following criteria:

1.Must be a randomized controlled trial (RCT).

2.The research must be about (Patients: solid tumors patients with CIT older than 18 years old; (3) Interventions: Etrombopag or Romiplostim or Avatrombopag compared with placebo or blank; (4) Outcome indicators: incidence of chemotherapy dose reduction or delays, bleeding events, platelet transfusion, incidence of grade 3 or 4 thrombocytopenia, incidence of platelet count400×109/L, adverse events(AEs), serious AEs, embolism events and deaths).

All other types of studies, including nonrandomized research designs, will be excluded.

23. Context.

Give summary details of the setting or other relevant characteristics, which help define the inclusion or exclusion criteria.

24. * Main outcome(s).

Give the pre-specified main (most important) outcomes of the review, including details of how the outcome is defined and measured and when these measurement are made, if these are part of the review inclusion criteria.

The main outcomes of our systematic review include the following:

1. Chemotherapy dose reduction or delay: We will assess whether thrombopoietin receptor agonists reduce the frequency of chemotherapy dose reduction or delay chemotherapy in chemotherapy-induced thrombocytopenia. This outcome will be assessed against chemotherapy dose reductions or delays reported in the included studies.

2.Platelet transfusions: We will investigate whether thrombopoietin receptor agonists reduce the need for platelet transfusions. This outcome will be assessed according to the platelet transfusion status reported in the included studies.

3.Incidence of Grade 3 or 4 Thrombocytopenia: We will assess whether thrombopoietin receptor agonists reduce the risk of developing Grade 3 or 4 thrombocytopenia. This outcome will be assessed against the reported incidence of thrombocytopenia in the included studies.

4.Bleeding events: We will investigate whether thrombopoietin receptor agonists can reduce the incidence of bleeding events. This includes evaluating various types of bleeding events such as bruising, nosebleeds, etc. Definitions of bleeding events will be assessed based on reports from included studies.

Measures of effect

Please specify the effect measure(s) for you main outcome(s) e.g. relative risks, odds ratios, risk difference, and/or 'number needed to treat.

25. * Additional outcome(s).

List the pre-specified additional outcomes of the review, with a similar level of detail to that required for main outcomes. Where there are no additional outcomes please state ‘None’ or ‘Not applicable’ as appropriate to the review

The dditional outcomes of our systematic review include the following:

1.Platelet count 400×10^9/L: We will evaluate the effect of thrombopoietin receptor agonists on platelet count in patients with solid tumors, with a particular focus on whether the platelet count reaches or exceeds the level of 400×10^9/L. This outcome will be assessed based on platelet count data reported in the included studies.

2.Adverse events (AEs): We will investigate adverse events that occurred during the treatment with thrombopoietin receptor agonists, including but not limited to nausea, vomiting, headache, etc. Definitions of adverse events will be assessed based on reports from included studies.

3.Serious adverse events (serious AEs): We will look at the incidence of serious adverse events that may require hospitalization, cause permanent damage, or be life-threatening. Definitions of serious adverse events will be assessed based on reports from included studies.

4.Thrombosis: We will assess the relationship between thrombopoietin receptor agonist therapy and the risk of thrombosis. Thrombosis will be assessed according to reports in the included studies.

5.Mortality: We will examine mortality associated with treatment with thrombopoietin receptor agonists. Mortality will be assessed based on reports from included studies.

Measures of effect

Please specify the effect measure(s) for you additional outcome(s) e.g. relative risks, odds ratios, risk difference, and/or 'number needed to treat.

26. * Data extraction (selection and coding).

Describe how studies will be selected for inclusion. State what data will be extracted or obtained. State how this will be done and recorded.

Study selection will be based on the following inclusion criteria:

Study design: We will only include randomized controlled trials (RCTs).

Participants: solid tumors patients with CIT older than 18 years old

Interventions: Etrombopag or Romiplostim or Avatrombopag compared with placebo or blank

Outcomes: Studies had to report our pre-determined primary and additional outcomes.

Two authors (CW and LYBX) extracted the data independently to complete the extraction table, disagreements between authors were resolved by discussion or decided by the third party. The date included in the extraction table were as follows: (1) first author's name, publication time, regions and registration number of trails, randomization, total number of participants; (2) age and gender of the patients; (3) intervention char-acteristics (type, dose, and duration); (4) outcome indicators: bleeding events, platelet transfusion, chemotherapy dose reduction or delays, incidence of grade 3 or 4 throm-bocytopenia, incidence of platelet count400×109/L; (5) safety data: AEs, serious AEs, thrombosis and mortality.

27. * Risk of bias (quality) assessment.

State which characteristics of the studies will be assessed and/or any formal risk of bias/quality assessment tools that will be used.

We will assess the following characteristics of included studies:

1. Method of random allocation: We will assess the method of random allocation in each RCT to determine whether there has been selection bias.

2. Covering of random allocation: We will assess whether adequate random allocation masking is used in the trial to reduce informed bias.

3. Patient selection: We will assess whether the included studies represent a representative patient population to reduce selection bias.

4. Intervention implementation: We will focus on the implementation of the intervention to determine whether there is implementation bias.

5. Assessment of outcomes: We will assess measurement and reporting of primary and additional outcomes to determine whether there is measurement bias.

6. Incomplete data: We checked="checked" value="1" each study for missing data and considered reporting bias.

To assess study quality and risk of bias, we will use the Cochrane Collaboration's risk of bias tool. This will include scoring specific items of risk of bias in each study in order to determine the overall quality and credibility of each study

28. * Strategy for data synthesis.

Describe the methods you plan to use to synthesise data. This **must not be generic text** but should be **specific to your review** and describe how the proposed approach will be applied to your data. If meta-analysis is planned, describe the models to be used, methods to explore statistical heterogeneity, and software package to be used.

We planned to use a meta-analysis approach to synthesize study results. First, we will extract the effect sizes and standard errors associated with our main and additional outcomes for each included study. If multiple studies reported the same effect, we calculated their weighted average effect.

For the meta-analysis, we planned to use a random-effects model, as we expected that there might be some degree of heterogeneity between studies. Random effects models will account for both within-study and between-study variability to estimate population effects. We will use the RevMan software recommended by the Cochrane Collaboration to perform the meta-analysis.

To explore statistical heterogeneity, we will perform the I^2 statistic to assess variability in study findings. If high levels of heterogeneity were found (I^2 50%), we performed subgroup analyzes to try to explain the source of the heterogeneity. In addition, if the data permit, we also plan to perform a sensitivity analysis to assess the impact of different inclusion criteria on the results.

In summary, we will use a meta-analysis approach to synthesize the effect sizes of the included studies, employ a random-effects model to account for heterogeneity, and use RevMan software to perform the

analysis

29. * Analysis of subgroups or subsets.

State any planned investigation of ‘subgroups’. Be clear and specific about which type of study or participant will be included in each group or covariate investigated. State the planned analytic approach.

We plan to perform subgroup analyses to study the effects of thrombopoietin receptor agonists in patients with different types of solid tumors. Below are the subgroups we plan to study and the planned analysis methods:

1. Solid Tumor Type Subgroups: we will divide our patients into subgroups of different solid tumor types, such as breast cancer, lung cancer, colorectal cancer, and so on. For each subgroup, we will analyze the efficacy and safety of thrombopoietin receptor agonists separately.

2. Chemotherapy regimen subgroups: we will consider different chemotherapy regimens that patients receive, such as different drug combinations or doses. We plan to analyze the effect of thrombopoietin receptor agonists in each chemotherapy regimen subgroup.

3. Patient characteristics subgroups: we will also consider patient characteristics such as age, gender, and baseline platelet count to determine if these factors have an impact on treatment effects.

For the subgroup analyses, we plan to use a random effects model to estimate the effect in each subgroup to account for possible heterogeneity. We will compare differences in effects between subgroups and perform relevant statistical tests. This will help to determine whether there are differences in the efficacy and safety of thrombopoietin receptor agonists in patients with different types of solid tumors, as well as with different chemotherapy regimens and patient characteristics

30. * Type and method of review.

Select the type of review, review method and health area from the lists below.

Type of review

Cost effectiveness
No

Diagnostic
No

Epidemiologic
No

Individual patient data (IPD) meta-analysis

No

Intervention

Yes

Living systematic review

No

Meta-analysis

Yes

Methodology

No

Narrative synthesis

No

Network meta-analysis

No

Pre-clinical

No

Prevention

No

Prognostic

No

Prospective meta-analysis (PMA)

No

Review of reviews

No

Service delivery

No

Synthesis of qualitative studies

No

Systematic review

Yes

Other

No

Health area of the review

Alcohol/substance misuse/abuse

No

Blood and immune system

No

Cancer
Yes

Cardiovascular
No

Care of the elderly
No

Child health
No

Complementary therapies
No

COVID-19
No

Crime and justice
No

Dental
No

Digestive system
No

Ear, nose and throat
No

Education
No

Endocrine and metabolic disorders
No

Eye disorders
No

General interest
No

Genetics
No

Health inequalities/health equity
No

Infections and infestations
No

International development

No

Mental health and behavioural conditions

No

Musculoskeletal

No

Neurological

No

Nursing

No

Obstetrics and gynaecology

No

Oral health

No

Palliative care

No

Perioperative care

No

Physiotherapy

No

Pregnancy and childbirth

No

Public health (including social determinants of health)

No

Rehabilitation

No

Respiratory disorders

No

Service delivery

No

Skin disorders

No

Social care

No

Surgery

No

Tropical Medicine

No

Urological

No

Wounds, injuries and accidents

No

Violence and abuse

No

31. Language.

Select each language individually to add it to the list below, use the bin icon to remove any added in error.

English

There is not an English language summary

32. * Country.

Select the country in which the review is being carried out. For multi-national collaborations select all the countries involved.

China

33. Other registration details.

Name any other organisation where the systematic review title or protocol is registered (e.g. Campbell, or The Joanna Briggs Institute) together with any unique identification number assigned by them. If extracted data will be stored and made available through a repository such as the Systematic Review Data Repository (SRDR), details and a link should be included here. If none, leave blank.

34. Reference and/or URL for published protocol.

If the protocol for this review is published provide details (authors, title and journal details, preferably in Vancouver format)

Add web link to the published protocol.

Or, upload your published protocol here in pdf format. Note that the upload will be publicly accessible.

Yes I give permission for this file to be made publicly available

Please note that the information required in the PROSPERO registration form must be completed in full even if access to a protocol is given.

35. Dissemination plans.

Do you intend to publish the review on completion?

No

Give brief details of plans for communicating review findings.?

36. Keywords.

Give words or phrases that best describe the review. Separate keywords with a semicolon or new line. Keywords help PROSPERO users find your review (keywords do not appear in the public record but are included in searches). Be as specific and precise as possible. Avoid acronyms and abbreviations unless these are in wide use.

37. Details of any existing review of the same topic by the same authors.

If you are registering an update of an existing review give details of the earlier versions and include a full bibliographic reference, if available.

38. * Current review status.

Update review status when the review is completed and when it is published.New registrations must be ongoing so this field is not editable for initial submission.

Please provide anticipated publication date

Review_Ongoing

39. Any additional information.

Provide any other information relevant to the registration of this review.

40. Details of final report/publication(s) or preprints if available.

Leave empty until publication details are available OR you have a link to a preprint (NOTE: this field is not editable for initial submission). List authors, title and journal details preferably in Vancouver format.

Give the link to the published review or preprint.
