# Supplementary material for: Efficacy and safety of thrombopoietin receptor agonists in solid tumors with chemotherapy-induced thrombocytopenia: a meta-analysis
Source: BMC Pharmacol Toxicol. 2023 Dec 1;24:71. doi: 10.1186/s40360-023-00707-5 (PMC10693054; doi:10.1186/s40360-023-00707-5)
Supplement: Supplementary file 3 — Supplementary Material 3 [file 40360_2023_707_MOESM3_ESM.docx]

This file includes detailed search strategies.

Search Terms and Search Strategies

Search Terms: Four parts

| thrombopoietin receptor agonists  TPO-RAs  Romiplostim  Eltrombopag  Avatrombopag  Lusutrombopag | Drug Therapy  Chemotherapies  Pharmacotherapies  Pharmacotherapy  Drug Therapies  Chemotherapy  Therapy, Drug  Therapies, Drug  Induction Chemotherapies  Chemotherapies, Induction  Chemotherapy, Induction  Chemotherapy, Consolidation  Consolidation Chemotherapies  Chemotherapies, Consolidation | thrombocytopenia  macrothrombocytopaenia  macrothrombocytopenia  platelet count decreased  platelet deficiency  thrombocyte deficiency  thrombocytopaenias  thrombopenia  thrombopenias | clinical trial  clinical drug trial  major clinical trial  trial, clinical  randomized controlled trial  controlled trial, randomized  randomised controlled study  randomised controlled trial  randomized controlled study  trial, randomized controlled  clinical study |
| --- | --- | --- | --- |

Search Strategies in Pubmed:

| (thrombopoietin receptor agonists [Title/Abstract]) OR (TPO-Ras[Title/Abstract]) OR (Romiplostim[Title/Abstract]) OR (Eltrombopag[Title/Abstract]) OR (Avatrombopag[Title/Abstract]) OR (lusutrombopag[Title/Abstract]) | (Drug Therapy[Mesh]) OR (Chemotherapies[Title/Abstract]) OR (Pharmacotherapies[Title/Abstract]) OR (Pharmacotherapy[Title/Abstract]) OR (Drug Therapies[Title/Abstract]) OR (Chemotherapy[Title/Abstract]) OR (Therapy, Drug[Title/Abstract]) OR (Therapies, Drug[Title/Abstract]) OR (Induction Chemotherapies[Title/Abstract]) OR (Chemotherapies, Induction[Title/Abstract]) OR (Chemotherapy, Induction[Title/Abstract]) OR (Chemotherapy, Consolidation[Title/Abstract]) OR (Consolidation Chemotherapies[Title/Abstract]) OR (Chemotherapies, Consolidation[Title/Abstract]) | (thrombocytopenia[Mesh]) OR macrothrombocytopaenia[Title/Abstract] OR macrothrombocytopenia[Title/Abstract] OR platelet count decreased[Title/Abstract] OR platelet deficiency[Title/Abstract] OR thrombocyte deficiency[Title/Abstract] OR thrombocytopaenias[Title/Abstract] OR thrombopenia[Title/Abstract] OR thrombopenias[Title/Abstract] | (clinical trial[Mesh]) OR (clinical drug trial[Title/Abstract]) OR (major clinical trial[Title/Abstract]) OR (trial, clinical[Title/Abstract]) OR (randomized controlled trial[Title/Abstract]) OR (controlled trial, randomized[Title/Abstract]) OR (randomised controlled study[Title/Abstract]) OR (randomised controlled trial[Title/Abstract]) OR (randomized controlled study[Title/Abstract]) OR (trial, randomized controlled[Title/Abstract]) OR (clinical study[Title/Abstract]) |
| --- | --- | --- | --- |

Search Strategies in Embase:

| ‘thrombopoietin receptor agonists‘:ab,ti OR ‘TPO-Ras’:ab,ti OR ‘Romiplostim’:ab,ti OR ‘Eltrombopag’:ab,ti OR ‘Avatrombopag’:ab,ti OR ‘lusutrombopag’:ab,ti | 'chemotherapy'/exp OR 'chemotherapies':ab,ti OR 'pharmacotherapies':ab,ti OR 'pharmacotherapy':ab,ti OR 'drug therapies':ab,ti OR 'chemotherapy':ab,ti OR 'therapy, drug':ab,ti OR 'therapies, drug':ab,ti OR 'induction chemotherapies':ab,ti OR 'chemotherapies, induction':ab,ti OR 'chemotherapy, induction':ab,ti OR 'chemotherapy, consolidation':ab,ti OR 'consolidation chemotherapies':ab,ti OR 'chemotherapies, consolidation':ab,ti | ‘thrombocytopenia’/exp OR ‘macrothrombocytopaenia’:ab,ti OR ‘macrothrombocytopenia’:ab,ti OR ‘platelet count decreased’:ab,ti OR ‘platelet deficiency’:ab,ti OR ‘thrombocyte deficiency’:ab,ti OR ‘thrombocytopaenias’:ab,ti OR ‘thrombopenia’:ab,ti OR ‘thrombopenias’:ab,ti | ‘clinical trial’/exp OR ‘clinical drug trial’:ab,ti OR ‘major clinical trial’:ab,ti OR ‘trial, clinical’:ab,ti OR ‘randomized controlled trial’:ab,ti OR ‘controlled trial, randomized’:ab,ti OR ‘randomised controlled study’:ab,ti OR ‘randomised controlled trial’:ab,ti OR ‘randomized controlled study’:ab,ti OR ‘trial, randomized controlled’:ab,ti OR ‘clinical study’:ab,ti |
| --- | --- | --- | --- |

Search Strategies in FMRS:

| (thrombopoietin receptor agonists[TIAB] OR TPO-Ras[TIAB] OR Romiplostim[TIAB] OR Eltrombopag[TIAB] OR Avatrombopag[TIAB] OR lusutrombopag[TIAB] ) | (Drug Therapy[mh]) OR (Chemotherapies[TIAB]) OR (Pharmacotherapies[TIAB]) OR (Pharmacotherapy[TIAB]) OR (Drug Therapies[TIAB]) OR (Chemotherapy[TIAB]) OR (Therapy, Drug[TIAB]) OR (Therapies, Drug[TIAB]) OR (Induction Chemotherapies[TIAB]) OR (Chemotherapies, Induction[TIAB]) OR (Chemotherapy, Induction[TIAB]) OR (Chemotherapy, Consolidation[TIAB]) OR (Consolidation Chemotherapies[TIAB]) OR (Chemotherapies, Consolidation[TIAB]) | (thrombocytopenia[mh]) OR (macrothrombocytopaenia[TIAB]) OR (macrothrombocytopenia[TIAB]) OR (platelet count decreased[TIAB]) OR (platelet deficiency[TIAB]) OR (thrombocyte deficiency[TIAB]) OR (thrombocytopaenias[TIAB]) OR (thrombopenia[TIAB]) OR (thrombopenias[TIAB]) | (clinical trial[mh])OR (clinical drug trial[TIAB])OR (major clinical trial[TIAB])OR (trial, clinical[TIAB])OR (randomized controlled trial[TIAB])OR (controlled trial, randomized[TIAB])OR (randomised controlled study[TIAB])OR (randomised controlled trial[TIAB])OR (randomized controlled study[TIAB])OR (trial, randomized controlled[TIAB])OR (clinical study[TIAB]) |
| --- | --- | --- | --- |

Search Strategies in Web of Science：

| (((((TS=(TPO-Ras)) OR TS=(thrombopoietin receptor agonists)) OR TS=(Romiplostim)) OR TS=(Eltrombopag)) OR TS=( Avatrombopag)) OR TS=(lusutrombopag ) | (((((((((((((TS=(Drug Therapy)) OR TS=(Chemotherapies)) OR TS=(Pharmacotherapies)) OR TS=(Pharmacotherapy)) OR TS=(Drug Therapies)) OR TS=(Chemotherapy)) OR TS=(Therapy, Drug)) OR TS=(Therapies, Drug)) OR TS=(Induction Chemotherapies)) OR TS=(Chemotherapies, Induction)) OR TS=(Chemotherapy, Induction)) OR TS=(Chemotherapy, Consolidation)) OR TS=(Consolidation Chemotherapies)) OR TS=(Chemotherapies, Consolidation) | ((((((((TS=(thrombocytopenia)) OR TS=(macrothrombocytopaenia)) OR TS=(macrothrombocytopenia)) OR TS=(platelet count decreased)) OR TS=(platelet deficiency)) OR TS=(thrombocyte deficiency)) OR TS=(thrombocytopaenias)) OR TS=(thrombopenia)) OR TS=(thrombopenias) | ((((((((((TS=(clinical trial)) OR TS=(clinical drug trial)) OR TS=(major clinical trial)) OR TS=(trial, clinical)) OR TS=(randomized controlled trial)) OR TS=(controlled trial, randomized)) OR TS=(randomised controlled study)) OR TS=(randomised controlled trial)) OR TS=(randomized controlled study)) OR TS=(trial, randomized controlled)) OR TS=(clinical study) |
| --- | --- | --- | --- |

Search Strategies in Cochrane:

| #1 (thrombopoietin receptor agonists):ti,ab,kw (Word variations have been searched)  #2 (TPO-RAs):ti,ab,kw (Word variations have been searched)  #3 (Romiplostim):ti,ab,kw (Word variations have been searched)  #4 (Eltrombopag):ti,ab,kw (Word variations have been searched)  #5 (Avatrombopag):ti,ab,kw (Word variations have been searched)  #6 (Lusutrombopag):ti,ab,kw (Word variations have been searched)  #7 #1 OR #2 OR #3 OR #4 OR #5 OR #6 | #1 MeSH descriptor: [Drug Therapy] explode all trees  #2 (chemotherapies):ti,ab,kw (Word variations have been searched)  #3 (Pharmacotherapies):ti,ab,kw (Word variations have been searched)  #4 (Pharmacotherapy):ti,ab,kw (Word variations have been searched)  #5 (Drug Therapies):ti,ab,kw (Word variations have been searched)  #6 (Chemotherapy):ti,ab,kw (Word variations have been searched)  #7 (Therapy, Drug):ti,ab,kw (Word variations have been searched)  #8 (Therapies, Drug):ti,ab,kw (Word variations have been searched)  #9 (Induction Chemotherapies):ti,ab,kw (Word variations have been searched)  #10 (Chemotherapies, Induction):ti,ab,kw (Word variations have been searched)  #11 (Chemotherapy, Induction):ti,ab,kw (Word variations have been searched)  #12 (Chemotherapy, Consolidation):ti,ab,kw (Word variations have been searched)  #13 (Consolidation Chemotherapies):ti,ab,kw (Word variations have been searched)  #14 (Chemotherapies, Consolidation):ti,ab,kw (Word variations have been searched)  #15 #1 OR #2 OR #3 OR #4 OR #5 OR #6 OR #7 OR #8 OR #9 OR #10 OR #11 OR #12 OR #13 OR #14 | #1 MeSH descriptor: [Thrombocytopenia] explode all trees  #2 (macrothrombocytopaenia):ti,ab,kw (Word variations have been searched)  #3 (macrothrombocytopenia):ti,ab,kw (Word variations have been searched)  #4 (platelet count decreased):ti,ab,kw (Word variations have been searched)  #5 (platelet deficiency):ti,ab,kw (Word variations have been searched)  #6 (thrombocyte deficiency):ti,ab,kw (Word variations have been searched)  #7 (thrombocytopaenias):ti,ab,kw (Word variations have been searched)  #8 (thrombopenia):ti,ab,kw (Word variations have been searched)  #9 (thrombopenias):ti,ab,kw (Word variations have been searched)  #10 #1 OR #2 OR #3 OR #4 OR #5 OR #6 OR #7 OR #8 OR #9 | #1 MeSH descriptor: [Clinical Trial] explode all trees  #2 (clinical drug trial):ti,ab,kw (Word variations have been searched)  #3 (major clinical trial):ti,ab,kw (Word variations have been searched)  #4 (trial, clinical):ti,ab,kw (Word variations have been searched)  #5 (randomized controlled trial):ti,ab,kw (Word variations have been searched)  #6 (controlled trial, randomized):ti,ab,kw (Word variations have been searched)  #7 (randomised controlled study):ti,ab,kw (Word variations have been searched)  #8 (clinical study):ti,ab,kw (Word variations have been searched)  #9 (trial, randomized controlled):ti,ab,kw (Word variations have been searched)  #10 #1 OR #2 OR #3 OR #4 OR #5 OR #6 OR #7 OR #8 OR #9 |
| --- | --- | --- | --- |
